# Modeling the long-range effect of an inversion downstream of *EFNB1* concludes a 43-year molecular diagnostic odyssey for craniofrontonasal syndrome

**DOI:** 10.1038/s41431-025-01887-w

**Published:** 2025-06-09

**Authors:** Dong Li, Leticia S. Matsuoka, Sarah Donoghue, Cuiping Hou, Alanna Strong, Donna M. McDonald-McGinn, Linton Whitaker, Jesse Taylor, Elizabeth J. Bhoj, Hakon Hakonarson, Elaine H. Zackai

**Affiliations:** 1 Center for Applied Genomics, The Children’s Hospital of Philadelphia, Philadelphia, PA USA; 2 Division of Human Genetics, The Children’s Hospital of Philadelphia, Philadelphia, PA USA; 3 Department of Pediatrics, University of Pennsylvania Perelman School of Medicine, Philadelphia, PA USA; 4 Division of Plastic Surgery, Department of Surgery, The Children’s Hospital of Philadelphia, Philadelphia, PA USA

**Keywords:** Disease genetics, Stem-cell research

## Abstract

Craniofrontonasal syndrome (CFNS; MIM #304110) is a rare craniofacial disorder characterized by hypertelorism, a broad nasal root with a bifid nasal tip, orofacial clefting, and genital malformations caused by pathogenic variants in the X-linked gene *EFNB1* (MIM *300035). CFNS exhibits sex-specific heterogeneity, with increased severity in females likely secondary to cellular interference related to random X-inactivation, resulting in mosaic *EFNB1* expression. Previous studies have identified over 140 variants in *EFNB1*, but approximately 20% of CFNS have negative molecular testing, either due to a yet undiscovered causal gene or causal variants in regulatory regions not covered by traditional genetic testing methodologies. Here, we report a two-generation family with a clinical diagnosis of CFNS and negative clinical molecular testing. Research short-read genome testing identified a 2-Mb inversion together with two smaller deletions (13- and 7-bp), about 106-Kb downstream of *EFNB1*, which cosegregated with CFNS. Patient-derived fibroblasts reprogrammed into induced pluripotent stem cells (iPSCs) demonstrated two distinct iPSC populations in affected females, where one or other of the two X chromosomes was inactivated. In vitro assays further demonstrated that iPSCs with the active X chromosome bearing the inversion, exhibited a significant increase in *EFNB1* expression, suggesting allelic imbalance contributes to mosaic *EFNB1* expression. These findings nominate a novel causal variant type of CFNS, conclude a 43-year diagnostic odyssey for an affected family, and offer new hope for family planning for affected individuals.

## Introduction

Craniofrontonasal syndrome (CFNS; MIM #304110) was initially described by two groups in 1979 (reference [1, 2]) as a craniofacial syndrome characterized by hypertelorism, a broad nasal root with a bifid nasal tip, orofacial clefting, dry, wooly hair, mild skeletal abnormalities, and genital malformations. Females often have more severe disease with multisystem involvement, in contrast to males, where hypertelorism can be the only presenting feature. In 2004, genetic variants in the X-linked gene *EFNB1* (MIM *300035), encoding the transmembrane signaling molecule ephrin-B1, were identified as the underlying cause of CFNS [3, 4].

This paradoxical difference in disease severity between males and females, termed cellular interference, was first characterized in 2004 and relates to random X-inactivation, which forms two distinct cell populations, each expressing either the wild-type or mutant allele [3–5]. The coexistence of these two cell populations subsequently gives rise to mosaic *EFNB1* expression, which disrupts cell adhesion and contributes to the abnormal tissue boundary formation. This hypothesis was supported by the subsequent identification of severely affected males carrying mosaic variants in the *EFNB1* gene [6] and robust cell segregation observed in mixed human neuroepithelial cells with mosaic *EFNB1* expression [7].

While previous studies have identified more than 140 causal variants in the *EFNB1* gene, including upstream noncoding [6, 8], nonsense, missense, frameshift, splice-altering, duplication [9] and deletion [10, 11] variants, approximately 20% of CFNS patients have negative molecular testing [10, 12, 13]. In this study, we report the discovery of a 2-Mb inversion, about 106-Kb downstream of *EFNB1*, cosegregating with CFNS in a two-generation pedigree revealed by short-read genome sequencing. To evaluate the pathogenicity of the inversion, we reprogrammed patient-derived fibroblasts and an X-inactivation assay was used to determine which X was active in each individual clone once isolated. We further demonstrated that cells bearing the inversion allele on the active X chromosome had a significant increase in *EFNB1* expression. These findings nominate a novel variant type as causal for CFNS, conclude a 43-year diagnostic odyssey for an affected family, and offer new hope for family planning for affected individuals.

## Materials and methods

### Genome sequencing and analysis

Whole genome sequencing was performed on four family members (I-2, II-1, II-4 and III-2) after negative exome (see Supplementary Materials and Methods) using an Illumina NovaSeq 6000 sequencer (Illumina, Inc., San Diego, CA, USA) with paired-end 100 bp reads. Libraries were generated from genomic DNA using the Illumina TruSeq DNA PCR-Free Library Prep Kit (Illumina, San Diego, CA). All the raw reads were aligned to the reference human genome using the Burrows-Wheeler Aligner [14] (BWA-Mem v0.7.12). BAM files were then processed by two short-read structural variant callers, Manta [15] and Wham [16], to capture structural variations (SVs) with default parameters. Similarly, the split and discordant reads files were generated by SpeedSeq [17] and were provided as inputs to Lumpy [18], another SV calling program. Confirmatory genotyping of the identified inversion was performed by polymerase chain reaction (PCR) and Sanger sequencing by using primers 5’-AAGGGGAGACTGGACTTCTTG-3’ and 5’-TCGAAGTCAGATCTCCAAATGT-3’, and 5’-TACCACCATTCAGACGGGAAA-3’ and 5’-AGGAGTTCAAGGCAGCAGT-3’. Thermal cycling condition was: 95 °C:5 m - (96 °C:5 s - 58 °C:5 s - 68 °C:40 s) × 35 - 72 °C:5 m - 4 °C:hold.

### Fibroblast reprograming to induced pluripotent stem cells (iPSCs) and molecular karyotyping

Fibroblasts from II-1 and II-4 were cultured following standard techniques. Fibroblasts were transduced using CytoTune-iPS 2.0 Sendai Reprogramming vectors containing polycistronic hKlf4–hOct3/4–hSox2 (hKOS), hcMyc, and hKlf4 (Thermo Fisher Scientific, Waltham, MA). Following transduction, cells were plated on tissue culture plates coated with Geltrex (Thermo Fisher Scientific, Waltham, MA) in Essential 8 media (Thermo Fisher Scientific, Waltham, MA). Once colonies began to form, they were picked using a pipet tip and media was switched to StemFlex (Thermo Fisher Scientific, Waltham, MA). To ensure no copy number variations (CNVs) occurred as a result of iPSC reprograming, DNA isolated from multiple colonies were genotyped with the Illumina Infinium Global Screening Array (llumina, San Diego, CA). The PennCNV algorithm [19] was used for CNV calling and aneuploidy was screened with GenomeStudio.

### X-chromosome inactivation (XCI) assay

From each of the original iPSC inductions, 20 separate individual clones were picked. Genomic DNA was isolated from each of these 20 clones. X-chromosome inactivation (XCI) analysis was performed at the androgen receptor (*AR*) triplet repeat locus, where the inactive X is methylated and, therefore, selectively retained after *HpaII* digestion. Following DNA methylation-sensitive restriction digestion with *HpaII* for each sample, the predigested and undigested samples were amplified by PCR. Fragment size data for digested and undigested pairs for each sample were collected using a capillary electrophoresis instrument to determine the XCI pattern [20].

### Real time quantitative reverse transcription PCR (RT-qPCR)

RT-qPCR was performed following the manufacturer’s protocol. For each sample/colony, RNA was extracted with using the RNeasy Mini Kit with on-column DNaseI digestion (Qiagen, catalog nos. 74104 and 79254), according to the manufacturer’s instructions and reverse transcription was done with the High Capacity cDNA Reverse Transcription Kit (Thermo Fisher Scientific, catalog no. 4368814) using random primers. Taqman assay primers (Thermofisher), *LIN28A, NANOG, OCT4 and SOX2* were used to check for iPSC markers expression and SeV, hKOS, hKlf4 and hcMyc primers (Thermo Fisher Scientific, Waltham, MA) were used to check for virus vector detection. Quantification of *EFNB1* expression was performed using the Fast SYBR Green PCR kit (Thermofisher). The data were analyzed by using the ΔΔct method, determined by RT-qPCR, and were normalized to *GAPDH* in same cDNA samples with values expressed as a percentage of the *GAPDH* control. The primers for *EFNB1* and *GAPDH* are 5’-CAATAGGCCAGAGCAGGAAATA-3’ and 5’- CTTCCATTGGATGTTGAGGTAATG-3’, and 5’-CTTTGGTATCGTGGAAGGACTC-3’ and 5’-AGTAGAGGCAGGGATGATGT-3’, respectively. Thermal cycler condition was: 95 °C:20 s - (95 °C:3 s - 60 °C:30 s) × 40.

### Western blotting of iPSC lysates

Whole cells were lysed in radioimmunoprecipitation assay (RIPA) buffer supplemented with protease inhibitors (complete protease inhibitor cocktail tablets, Roche, Mannheim, Germany) and phosphatase inhibitors sodium fluoride and sodium metavanadate. Approximately 60 micrograms of protein were separated on NuPAGE 4–12% Bis–Tris gels run with MOPS SDS running buffer (Life Technologies, Carlsbad, CA). Proteins were transferred to nitrocellulose membranes (Bio-Rad, Hercules, CA), and membranes were blocked with 5% bovine serum albumin (BSA; Thermofisher) in phosphate buffered saline containing 0.1% tween20 (PBST) for 30 min at room temperature. Membranes were incubated with primary antibodies diluted in 5% BSA in TBST [goat anti-Ephrin-B1(R&D Systems, Minneapolis, MN, catalog number AF473, 500 ng/ml final concentration); mouse anti-beta-actin (Santa Cruz, catalog number AC-15, 500 ng/ml final concentration)] overnight at 4 °C. Following 3 washes (5 minutes each) with PBST, membranes were incubated with horse radish peroxidase-conjugated donkey-anti-goat (R&D Systems, catalog number HAF017) or horse-anti-mouse (Cell Signaling Technology, Danvers, MA; catalog number 7076) secondary antibody diluted 1:10,000 in PBST for 30 min at room temperature. Following 3 more washes with PBST, western blots were developed using SuperSignal West Pico PLUS Chemiluminescent Substrate (ThermoFisher) and imaged using a KwikQuant Imager (Kindle Biosciences, Greenwich CT).

## Results

### Clinical report and clinical diagnostic studies

**Fig. 1:**
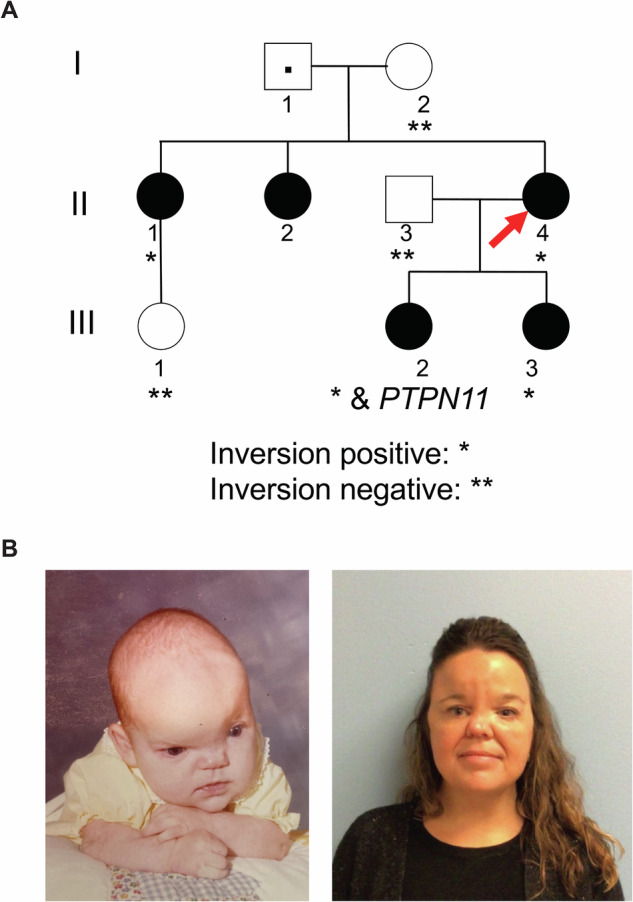
Pedigree and facial photographs showing craniofrontonasal syndrome. **A** Pedigree and cosegregation analysis. **B** Facial photographs of individual II-1 at 10 months old (before surgical repairs) and 46 years old (post synostosis and hypertelorism repairs), respectively.

The proband (II-4; Fig. 1A), now 43 years old, was first seen in 1980 at 9 months of age by Clinical Genetics, presenting with right coronal synostosis, hypertelorism, and a bifid nose. Family history was notable for one sister (II-1; Fig. 1B) with left coronal synostosis, hypertelorism, short columella, and a flat nasal tip noted at 4 years of age and a second sister (II-2) with a nasal cleft and hypertelorism without craniosynostosis. All three siblings were diagnosed clinically with CFNS. Their father (I-2) was noted to have hypertelorism and a slight widow’s peak but was otherwise unaffected. Both of the proband’s daughters (III-2 and III-3) were diagnosed clinically with CFNS. The clinical course for individual III-2 is additionally complicated by short stature, poor weight gain, strabismus, pulmonary stenosis and vascular ring (Fig. 1A). Genetic testing, including a karyotype, microarray, MLPA study for 22q11.2 deletion, and *EFNB1* gene sequencing and deletion/duplication tests, was negative.

### Additional molecular investigations with exome and genome sequencing

**Fig. 2:**
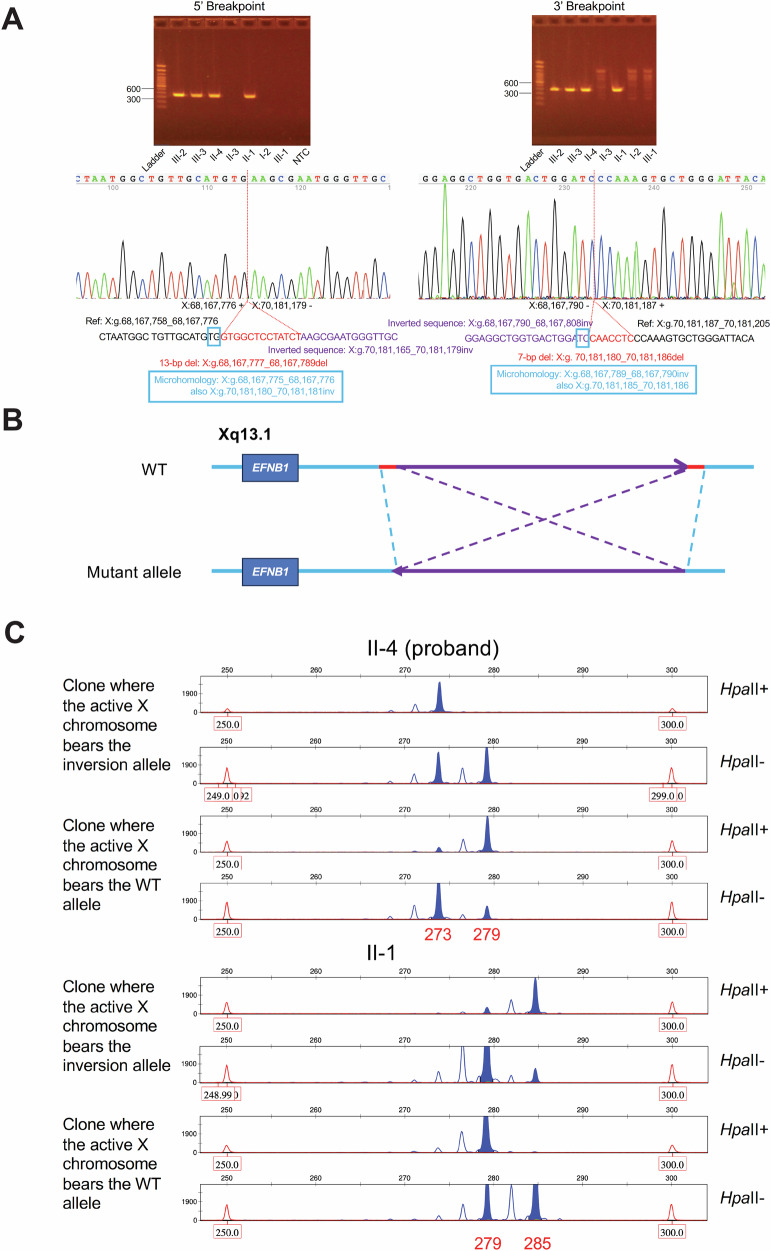
Sanger confirmation of identified inversion and two distinct iPSC populations revealed by DNA fragment analysis. **A** Sanger sequencing precisely mapped the breakpoints and confirmed the cosegregation pattern. **B** Simplified diagram showed the inversion together with two smaller deletions at both ends. **C** X-inactivation analysis in two affected family members (II-4 [proband) and II-1) revealed that each individual has two distinct cell populations. By analyzing *AR* locus fragment sizes as markers and recognizing that both individuals inherited the same X chromosome from their father, the allele with a fragment size of 279 was determined to harbor the *EFNB1* inversion (also see Supplementary Fig. 1).

Exome sequencing followed by Sanger validation in Individual III-2 revealed a de novo pathogenic *PTPN11* variant (c.236A>G, p.(Gln79Arg)), consistent with Noonan syndrome, accounting for her history of short stature, pulmonic stenosis, and failure to thrive, but not her CFNS clinical diagnosis (Fig. 1A). To identify the molecular basis of the CFNS phenotype, we performed genome sequencing, which revealed a heterozygous 2-Mb inversion at Xq13.1, about 106-Kb downstream of the *EFNB1* gene, captured by Lumpy with support from Manta. Follow-up PCR and Sanger sequencing determined that the inversion spanned from chrX(GRCh37):g.68,167,790 to chrX(GRCh37):g.70,181,179 with 2-bp microhomology sequences at both breakpoints (NC_000023.10:g.68167790_70181179inv; Fig. 2A, B). Interestingly, two small deletions were also identified at both ends of the inversion: NC_000023.10:g.68167777_68167789del (13-bp) and NC_000023.10:g.70181180_70181186del (7-bp; Fig. 2A, B). Query of the ENCODE dataset [21] demonstrated that the 5’ end of the inversion and the 13-bp deletion overlap with a *cis*-regulatory element (ccRE; ENCODE #: EH38E2757328). This ccRE has maximum DNase, H3K4me3, H3K27ac, and CTCF Z scores of 3.50, 1.19, 1.17, 3.48, respectively, suggesting that it possesses a CTCF-bound insulator property.

### Characterization of the impact of the inversion downstream of *EFNB1* in patient-derived iPSCs

**Fig. 3:**
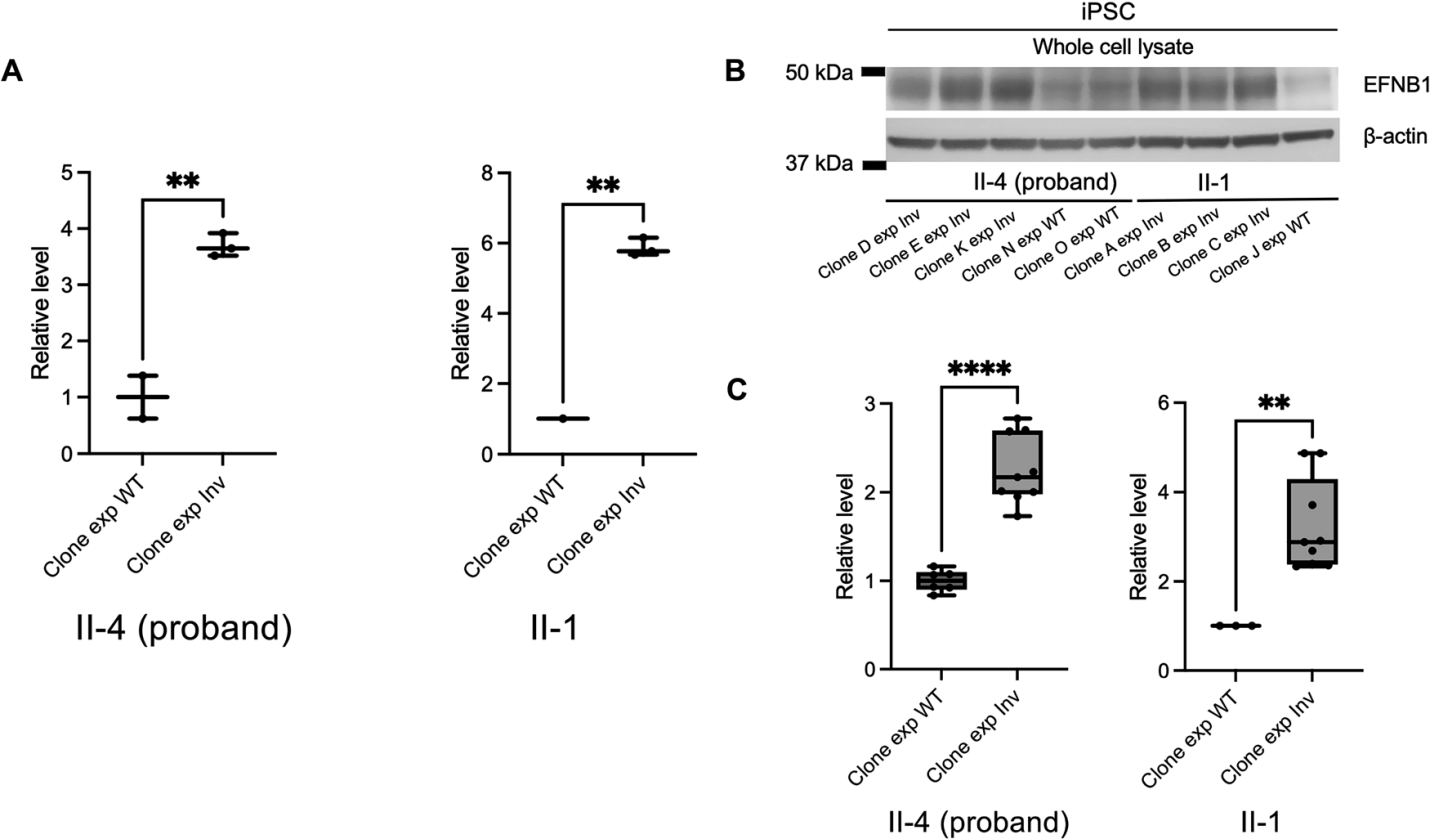
EFNB1 expression studies in iPSCs. **A** RT-qPCR analysis showed significantly increased *EFNB1* expression in clones in which the active X chromosome bears the inversion compared to clones in which active X chromosome bears the wildtype allele in both II-4 and II-1. Normalized values are illustrated by the box-and-whisker plot with all values. **P < 0.01 by two-tailed, unpaired *t* test. **B** Representative Western blot image confirmed the reduced EFNB1 level in both individuals. **C** Quantification analysis of Western blot. Normalized valued are illustrated by the box-and-whisker plot with all values. **P < 0.01; ****P < 0.0001 by two-tailed, unpaired *t* test.

Given the overlap, we hypothesize that the identified inversion and 13-bp deletion telomeric to *EFNB1* may disrupt a three-dimensional CTCF-bound topologically associated domain (TAD), altering *EFNB1* expression and contributing to allelic imbalance. To test this hypothesis, we obtained skin biopsies from two affected family members (II-1 and II-4) and reprogrammed the fibroblasts to generate stem cell lines. Genomic integrity assessment of all studied clones was assessed using the Infinium Global Screening Array, which revealed no copy number variations and no aneuploidy of any chromosomes. The iPSC lines generated in this study exhibited typical hallmarks of pluripotency, including the expression of endogenous pluripotency genes (data not shown). X-inactivation analysis was performed on 20 separate individual clones from each of the original iPSC inductions and revealed two cell populations with different inactivated X chromosomes (Fig. 2C; Supplementary Table 1). By analyzing fragment sizes and knowing that both individuals inherited the same X chromosome from their father, we were able to identify the clones bearing the inversion allele on the active X chromosome in the two affected individuals (Fig. 2C; Supplementary Fig. 1). Subsequently, RT-qPCR demonstrated significantly increased *EFNB1* expression in cells bearing the inversion allele on the active X chromosome in both individuals (Fig. 3A). This increase in *EFNB1* expression was further validated by Western blot analysis of iPSC whole cell lysates (Fig. 3B, C). Together, these data suggest that the inversion regulates EFNB1 expression through a long-range effect, leading to a relatively higher *EFNB1* expression level.

## Discussion

While deletions and duplications involving the *EFNB1* gene have previously been identified as causes for CFNS, the occurrence of an inversion involving only the regulatory region has not, to our knowledge, been described in CFNS. While this work was in progress, a patient with CFNS and a pericentric inversion involving the entire *EFNB1* gene was reported [22]. It was proposed that the pericentric inversion disrupts interaction with a specific enhancer (GH0XJ068989) located ~150 kb downstream of *EFNB1*, within the inverted region identified in our study. However, the observed increase in EFNB1 expression levels suggests that the enhancer landscape may be more complex than previously thought. As multiple candidate enhancers exist [22], further investigation will be needed to pinpoint the functional enhancer(s) for *EFNB1*. Recently, genome sequencing has emerged as comprehensive single-test approach in molecular diagnosis, offering several advantages [23–28], including improved the variant calling of both small variants and large structural variations due to its more uniform coverage across the entire genome compared to exome sequencing. Here, we illustrate its molecular diagnostic utility in a two-generation pedigree, following a 43-year observational period and extensive genetic investigations.

CFNS represents one of the three disorders where females harboring pathogenic heterozygous variants are more severely affected than males who carry hemizygous variants. The other two examples are *PCDH19*-related neurodevelopmental disorder [29] (MIM #300088) and *ARR3*-related early-onset high myopia [30] (MIM #301010). The suspected underlying mechanism driving this disparity is that random X-inactivation in heterozygous females produces a mixture of two cell populations [29, 31], causing patchy or mosaic gene expression that disrupts cell-cell communication and adhesion, ultimately leading to the aberrant tissue boundary formation. This cellular interference model is supported for *EFNB1* by animal studies, which demonstrate that female mice with heterozygous *Efnb1* deficiency have a more severe phenotype than either hemizygous male or homozygous KO female mice [32].

Given the overlap between the identified rearrangement and a ccRE (E2757328; chrX(GRCh37):g.68,167,666_68,167,985), which was classified CTCF-only/CTCF-bound by the ENCODE project based on high DNase and CTCF scores, along with low H3K4me3 and H3K27ac scores, we predicted that the rearrangement might disrupt or relocate the CTCF binding site in a different orientation, potentially disrupting the formation of a TAD or creating an alternative TAD loop. Several studies have shown that inversion of CTCF-binding sites can disrupt chromosomal loops, which in turn leads to increased or decreased gene expression [33, 34]. We initially hypothesized that the X chromosome expressing the identified rearrangement would reduce EFNB1 expression levels; however, contrary to our expectation, it led to elevated *EFNB1* mRNA and EFNB1 protein levels compared to the normal X chromosome. Interestingly, duplications of the *EFNB1* region have been previously reported in patients with hypertelorism [9, 35], and it has been shown that the X chromosome bearing the *EFNB1* duplication produces more *EFNB1* transcript than the normal X chromosome [9]. Our results further support the idea that an allelic imbalance in *EFNB1* expression could lead to a phenotype consistent with loss-of-function *EFNB1* variants, even though the detailed mechanism remains elusive.

Our study also demonstrates the importance of broad based testing to identify a dual diagnosis. Specifically, exome sequencing for subject III-2 unexpectedly identified a de novo pathogenic *PTPN11* variant. This variant explains her additional clinical presentations, including heart defects and poor weight gain. This highlights the significance of analyzing each affected family member individually when conducting genetic analysis. In this family, when four samples were analyzed collectively under the assumption of either X-linked or autosomal dominant inheritance pattern, the pathogenic *PTPN11* variant would not have been identified. A clinical diagnosis of Noonan syndrome would have been difficult, given the blended dysmorphology of Noonan syndrome and CFNS. The Noonan syndrome diagnosis had significant implications for her clinical care. Notably, she experienced substantial blood loss during a previous craniosynostosis surgery (documented at ~37cc/kg) and menorrhagia. Upon receiving a Noonan syndrome diagnosis, she was evaluated by Hematology and found to have mild factor VII, IX, and XI deficiencies, underscoring the necessity for hemostasis planning prior to any procedures in the future.

Over several years, the proband (II-4) had two children who are similarly affected with CFNS. If this specific inversion downstream of *EFNB1* had been identified early on, it could have provided reproductive options and enhanced decision-marking.

In summary, our study serves a compelling reminder of the necessity of comprehensive genetic analysis to uncover concealed genetic variants. Coupling with the analysis of long-range effects of the rearrangement in patient-derived iPSCs, our study brings closure to a 43-year-long molecular diagnostic journey for CFNS.

## Supplementary information

Supplemental material

Supplementary Table 1

## Data Availability

The datasets generated and/or analyzed during the current study are available from the corresponding author on reasonable request.
