# Supplementary material for: Modeling the long-range effect of an inversion downstream of EFNB1 concludes a 43-year molecular diagnostic odyssey for craniofrontonasal syndrome
Source: Eur J Hum Genet. 2025 Jun 9;33(12):1684–9. doi: 10.1038/s41431-025-01887-w (PMC12669737; doi:10.1038/s41431-025-01887-w)
Supplement: Supplementary file 1 — Supplemental material [file 41431_2025_1887_MOESM1_ESM.pdf]

SUPPLEMENTARY MATERIALS AND METHODS

Exome sequencing and analysis

Under an Institutional Review Board approved protocol (IRB #: 06-004886), informed consent was obtained from the patient's family. Permission for clinical photographs was given separately. Exome sequencing was performed on four family members (I-2, II-1, II-4 and III-2). The sequencing libraries were prepared using the Agilent SureSelect Human All Exon kit V5. Sequencing was performed on an Illumina HiSeq2500 sequencer (Illumina, Inc., San Diego, CA, USA) at Center for Applied Genomics (CAG) at The Children's Hospital of Philadelphia (CHOP). Data were quality controlled and analyzed using a custom-built pipeline that incorporates BWA-mem v0.7.12 for alignment, Picard v1.97 for PCR duplication removal, and GATK v2.6.5 for variant calling. ANNOVAR and SnpEff were used to functionally annotate the variants and collect minor allele frequency (MAF) data from 1,000 Genomes Projects, ESP6500SI, ExAC, gnomAD, and Kaviar. Relatively common variants for autosomal dominant or recessive modes of inheritance were excluded based on MAF threshold of 1% in either population dataset, and functional annotation, such as, synonymous, non-exonic, and non-splicing-altering. Subsequent gene prioritization was performed on the basis of deleterious prediction and biological relevance by referring to the Online Mendelian Inheritance in Man (OMIM) database and Human Gene Mutation Database (HGMD).

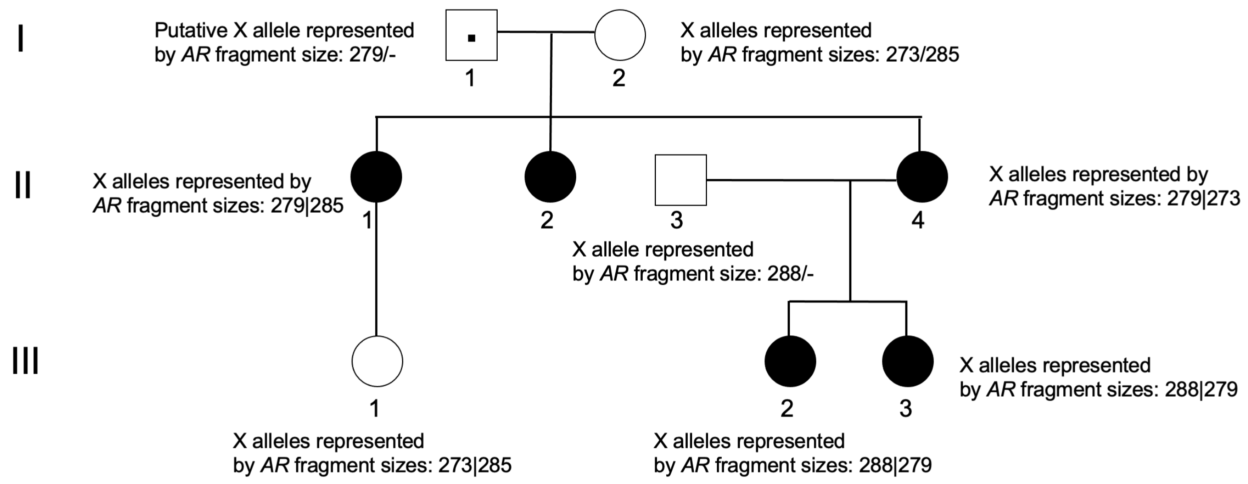

Figure S1. The phase relationship between the *AR* locus alleles and *EFNB1* inversion allele. Based on fragment size analysis in the X-inactivation assay with the *AR* triplet repeat locus from blood genomic DNA, and considering that the father is an obligate carrier, the *AR* allele with a fragment size of 279 was determined to be in phase with the *EFNB1* inversion.
